# “Not just another meta‐analysis”: Sources of heterogeneity in psychosocial treatment effect on cancer survival

**DOI:** 10.1002/cam4.1895

**Published:** 2019-01-01

**Authors:** Spela Mirosevic, Booil Jo, Helena C. Kraemer, Mona Ershadi, Eric Neri, David Spiegel

**Affiliations:** ^1^ Department of Family Medicine, Faculty of Medicine Ljubljana University of Ljubljana Ljubljana Slovenia; ^2^ Department of Psychiatry and Behavioral Sciences Stanford University School of Medicine Stanford California

**Keywords:** marital status, meta‐analysis, neoplasms, psychosocial support systems, survival

## Abstract

**Background:**

Currently, there are eight meta‐analyses that address the question whether psychosocial intervention can prolong survival with widely disparate conclusions. One reason for inconsistent findings may be the methods by which previous meta‐analyses were conducted.

**Methods:**

Databases were searched to identify valid randomized controlled trials that compared psychosocial intervention with usual care. Hazard ratios (HRs) and their confidence intervals were pooled to estimate the strength of the treatment effect on survival time, and *z*‐tests were performed to assess possible heterogeneity of effect sizes associated with different patient and treatment characteristics.

**Results:**

Twelve trials involving 2439 cancer patients that met screening criteria were included. The overall effect favored the treatment group with a HR of 0.71 (95% Cl 0.58‐0.88; *P *= 0.002). An effect size favoring treatment group was observed in studies sampling lower vs higher percentage of married patients’ (NNT = 4.3 vs NNT = 15.4), when Cognitive‐Behavioral Therapy was applied at early vs late cancer stage (NNT = 2.3 vs NNT = −28.6), and among patients’ older vs younger than 50 (NNT = 4.2 vs NNT = −20.5).

**Conclusions:**

Psychosocial interventions may have an important effect on survival. Reviewed interventions appear to be more effective in unmarried patients, patients who are older, and those with an early cancer stage who attend CBT. Limitations of previous meta‐analysis are discussed.

## 1 INTRODUCTION

Although the field of psychosocial oncology is relatively new, psychosocial interventions in cancer care are no longer rare. Helping patients to better cope with uncertainty about the future, fear of progression and death, and the wide range of cancer‐ and treatment‐related burdens is of growing importance in cancer survivorship.

The “gold standard” method to assess the efficacy of any treatment is the randomized controlled trial (RCT). With randomization, any differences in outcomes between treatments can be attributed to the intervention,^1^ rather than to selection bias or to statistical artifacts like regression to the mean or expectation effects. Past reviews and meta‐analyses of RCTs indicate that psychosocial interventions provide consistent psychological benefit for cancer patients.^2^, ^3^, ^4^ However, evidence that they also affect cancer survival is less consistent.

To date, there have been eight meta‐analyses on the issue of the efficacy of psychosocial interventions on cancer survival with widely disparate conclusions.^3^, ^4^, ^5^, ^6^, ^7^, ^8^, ^9^, ^10^ In 2004, two separate meta‐analyses by Chow et al^5^ and Smedslund and Ringdal^6^ addressed this issue and detected no overall treatment effects. Chow et al^5^ analyzed patients with various cancer histologies and various psychosocial treatments, and found no statistically significant difference in the overall survival rates at 1 and 4 years (example for 1‐year: Risk Ratio: RR = 0.94; 95% Cl [0.72‐1.22], *P* = 0.06). Edwards et al in 2008^7^ included five studies of group therapy for women with metastatic breast cancer (MBC) and concluded no overall effect at 1, 5, and 10 years (example for 5‐years: Odds Ratio: OR = 0.83; 95% Cl [0.53‐1.28], *P* = 0.4). In a 2013 Cochrane review, Mustafa et al^4^ analyzed only studies involving MBC patients and separated interventions by supportive‐expressive group therapy (SEGT) and cognitive‐behavioral therapy (CBT). The authors concluded that psychosocial interventions can be beneficial for survival in MBC, but only at 12 months (OR = 1.46; 95% Cl [1.07‐1.99], *P* = 0.02). Xia et al (2014)^8^ reported a significant survival difference at two years’ follow‐up (RR = 0.85; 95% CI [0.75‐0.96], *P* = 0.01) and two more recent meta‐analyses, by Jassim,^3^ and Shin and Kim,^9^ in 2015, concluded that psychosocial intervention was not associated with better survival (Hazard Ratio: HR = 0.76, 95% Cl [0.25‐2.32], *P* = 0.63 and HR = 0.83; 95% Cl [0.68‐1.10], *P* = 0.06, respectively); however, a subgroup analysis from Shin and Kim^9^ reported a significant effect for non‐metastatic patients and in interventions when health staff delivered the support. In 2016, the most recent meta‐analysis was conducted by Fu et al^10^ and reported a significant survival benefit at 1 and 2 years (example for 1‐year: RR = 0.82; 95% Cl [0.67‐1.00], *P* = 0.04), but not at four years (RR = 0.94; 95% Cl [0.85‐1.04], *P* = 0.24). Although a subgroup analysis was performed to compare group vs individual, a significant survival benefit was found for group but not individual interventions and only at one year (RR  = 0.57; 95% Cl [0.41‐0.79]).

Given this variation in conclusions from a presumably definitive analysis of data across studies, it is reasonable to ask whether meta‐analysis can be a meaningful statistical tool to compare psychological interventions?^11^ The problem lies not in whether meta‐analysis can be used, but in how meta‐analyses are conducted. One important criticism of meta‐analyses has been referred to as the “garbage in, garbage out” problem, referring to the quality of studies analyzed. Inclusion of studies of questionable validity to address the research question of interest can only confound meta‐analysis results. For example, including non‐randomized or non‐controlled trials along with RCTs is problematic. Including RCTs that drop subjects after randomization or cross patients from the randomly assigned treatment to the other, that is not doing analysis “by intention to treat,” is also unacceptable. Including RCTs in which there was either no clear protocol for delivery of psychosocial treatment or control, or where the clinicians delivering the treatment did not deliver the protocol‐driven treatment with fidelity, also creates problems.

Another problem of the meta‐analyses is the effect size used. As seen above, various studies have used the Odds Ratio (OR), the Risk Ratio (RR), or the Hazard Ratio (HR) as the effect size. The effect size used should be interpretable to clinicians and patients, but none of these effect sizes clearly convey clinical significance. The only commonality among the three is that they are all ratios; they do not estimate the same population parameter. The OR and RR are often misleading in survival analysis, as both of those use the proportions surviving at one selected time point (fixed follow‐up time), and the results at different time points may differ. The HR has major advantages over the RR or OR, in that it considers all observed survival times. As a result, the power to detect treatment efficacy is greater. Finally, when valid, HR can be translated into an effect size meaningful to clinicians: Number Needed to Treat (NNT). However, the validity of HR depends on the Proportional Hazards (PH) assumption, meaning in these types of survival analyses that the risk of mortality, absent the intervention being evaluated, is similar in the treatment and control groups over time, an assumption that covers many situations, but not all. Overall, HR is the preferred effect size for comparison of survival times.

Another important problem is heterogeneity, that is when the conclusions of valid studies are not consistent with each other. The purpose of a meta‐analysis of RCTs is to estimate the extent to which—in our case—psychosocial intervention *generally* influences cancer survival. However, meta‐analysis often face the “apples and oranges” problem, when studies addressing fundamentally different research questions (eg, different psychotherapies, different populations) are treated as if they dealt with the same research question. Some meta‐analyses have mixed very different treatments under the aegis of “psycho‐social”. Some RCTs used treatment as usual as the control group, others either an inactive or active placebo, and yet others an active comparator treatment (eg, education), but all are included in the same meta‐analysis. Meta‐analyses might also include RCTs using different inclusion/exclusion criteria, thus including studies of very different populations. Mixing different psychotherapies, different control conditions, or different populations produces the type of confusion seen in this context.

We conducted a systematic meta‐analysis with the aims of (a) evaluating the overall effect of psychosocial interventions on cancer survival and (b) exploring reasons for heterogeneity in results. The aim is to discern the most profitable directions of future research, and the approaches most likely to provide crucial information suitable for clinical decision‐making.

## 2 MATERIALS AND METHODS

The current paper was performed in accordance with recommendations from the Cochrane Collaboration using the Preferred Reporting Items for Systematic Review and Meta‐Analysis (PRISMA) statement.^12^

### 2.1 Search methods and eligibility criteria

**Figure 1:**
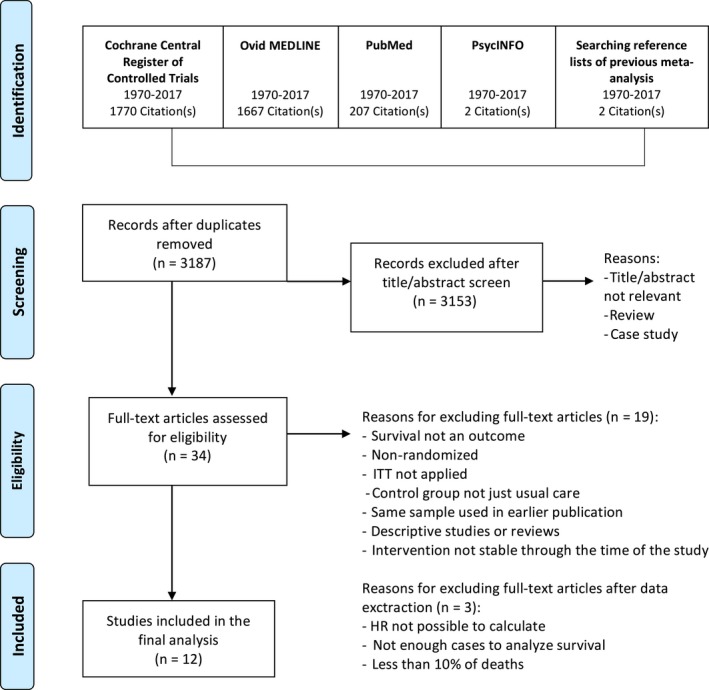
Flow chart of study selection performed on four stages

We systematically screened the literature through four electronic databases between September and December 2015 and regularly updated it through May of 2018 (see Table S1 for a specified search strategy). Moreover, we have also performed a hand‐search through the reference lists of previous meta‐analyses (Figure 1). All RCTs that compared survival benefits of psychosocial intervention in patients older than 18 years with any cancer histology were eligible for inclusion. Eligible trials were required to use only the usual standard of care as a control condition. The primary outcome was overall survival time, which was considered time from study entry until death. Only RTCs analyzed using the intention to treat principle were considered eligible. The effect size to be used was HR [rescaled as ln(HR) for certain computations] and as NNT to facilitate clinical interpretation of results. Studies without sufficient data to estimate HR (β and SE or Kaplan‐Meier plot)^13^ were excluded. Inclusion was based exclusively on meeting each of these objective criteria.

### 2.2 Study selection and data extraction

Study selection was performed in four stages (see Figure 1). At the first stage, studies identified through electronic databases and a backwards organic search from previous meta‐analyses were imported into a reference management database, EndNote X8. After removing duplicates, studies were screened using titles/abstracts. Only studies that were clearly not eligible (eg, not relevant, without appropriate methodology) were excluded in the second stage. At the third stage, two authors independently screened full‐text to determine which studies should be further included based on the predetermined criteria (see Eligibility Criteria). The follow‐up time, HR, and confidence intervals (CI) were extracted or calculated using a summary estimates.

### 2.3 Risk of bias assessment

Two authors assessed each study for methodological qualities by using adapted Cochrane risk of bias tool.^14^ Studies that met our eligibility criteria were reviewed on all of the suggested sources of bias, except the one involving performance and detection bias. Bias related to blinding participants and personnel (ie, performance bias) was not used, because it is often not possible to achieve in psychosocial interventions,^15^ and blinding of outcome assessment (ie, detection bias) was also of unlikely relevance because the only outcome was survival time. The research group discussed each study and resolved any of the potential disagreements.

### 2.4 Effect sizes’ transformation

We first obtained HRs (null value 1, range 0 to infinity) from included studies and assume that the PH assumption fits these situations reasonably well. Confidence intervals were computed and reported for ln(HR) (null value 0, range from minus to plus infinity) rather than HR, for mathematical convenience. NNT, a clinical significance indicator, is the number of pairs of patients, one sampled from the treatment group and the other from the control group, that would need to be sampled to expect to find one more pair for which the treatment survival is longer than the control survival (null value infinity, approaching 1 as treatment effectiveness increases).^16^ NNT can be compared to a commonly used effect size Cohen's d that is usually used for normally distributed outcomes. By Cohen's standards, an NNT of 9 is a small effect size, an NNT of 4 is a medium effect size, and an NNT of 3 is a large effect size.^17^

### 2.5 Identifying potential sources of heterogeneity

Of interest as possible sources of heterogeneity are demographic characteristics such as age and marital status; clinical characteristics such as type and stage of the cancer; intervention characteristics included type of intervention (ie, SEGT, CBT, psychoeducational, and supportive), format, dose, and duration.

The search here is at the population level, not at the level of the individual patient within the population. Moderators of treatment outcome are characteristics of individual patients that identify those who respond differently to the treatment, useful for personalized medical decisions. Sources of heterogeneity at the population level may or may not be moderators of treatment outcome at the individual level, due to a phenomenon long known as Simpson's Paradox, in which significant findings in subgroups may disappear when the groups are combined. Conversely, the Ecological Fallacy is also a problem, since it involves the assumption that characteristics of individuals can be well predicted by those of a group to which they belong. However, sources of heterogeneity at the population level may provide clues as to what characteristics might be considered as possible moderators of treatment outcome and inform choice of sites or inclusion/exclusion criteria in future studies.

### 2.6 Data analysis

Sources of heterogeneity analyses were conducted using a fixed effects model.^18^ After pooling the mean effect size for each group with similar characteristics, standard errors (SE) and confidence intervals were computed for ln(HR). Within every subgroup analysis, a heterogeneity analysis was performed. Finally, mean effect sizes between the two subgroups (eg, younger vs older patients) were compared.

A random effects model using the DerSimonian and Laird method,^19^ which assumes that the effect size varies among studies and that the studies represent a random sample of effect sizes, was also employed for the overall effect. We constructed a funnel plot to visually explore inconsistencies across studies as measured by relative symmetry^20^ and a sensitivity analysis to test the robustness of the findings, using jackknife analysis.^21^

**Figure 2:**
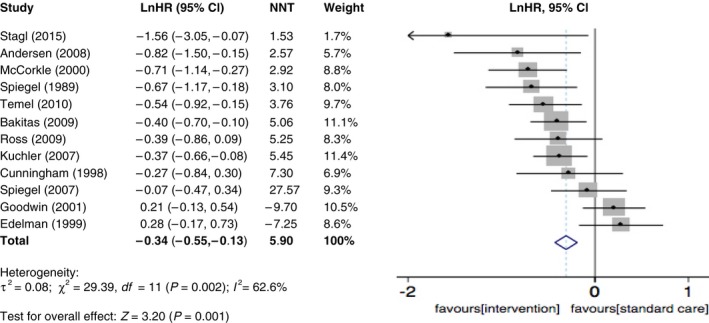
Forest plots of effects size for RCT studies of psychosocial intervention on overall survival

**Figure 3:**
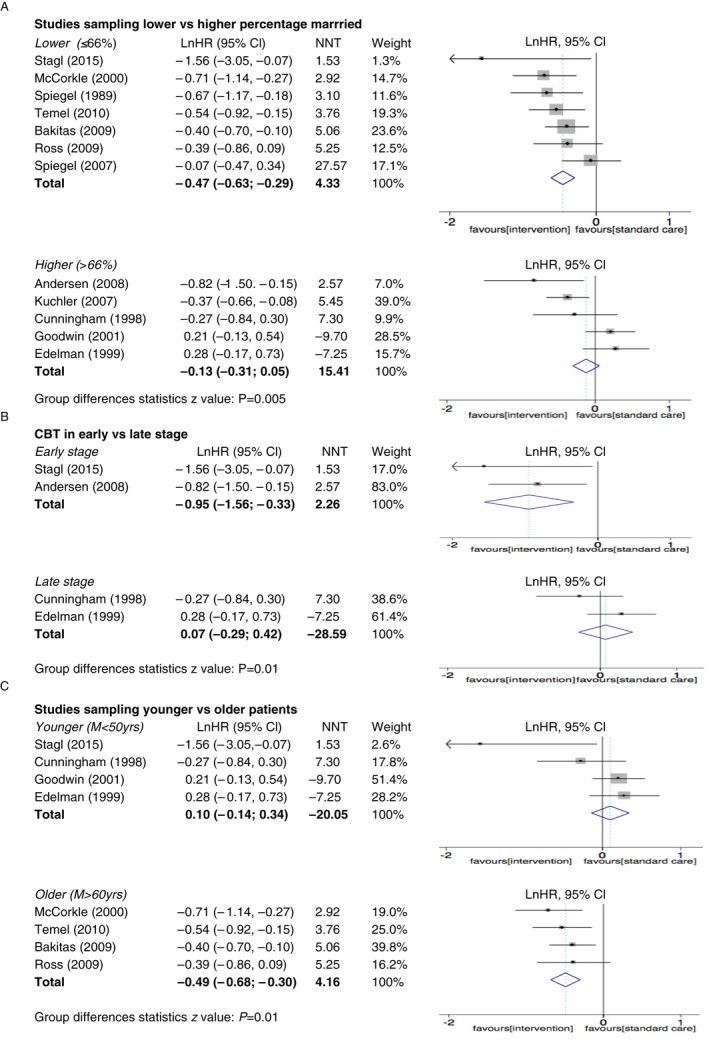
Forest plots of effects sizes for RCT studies of psychosocial interventions on survival. (A) Studies sampling lower vs higher percentage married, (B) CBT in early vs late stage, (C) studies sampling younger vs older patients

Disparity of the sample sizes can have a major impact on the number of studies needed to have to say something about the conclusion. In computing the pooled effect size and its standard error, weights were applied to individual studies that reflected their sample sizes (see Figures 2 and 3 “weight”).

## 3 RESULTS

The search strategy resulted in 3646 publications (Figure 1). After exclusion of duplicates and articles that were clearly not eligible for the analysis, 34 full‐text articles remained eligible for further analysis. Of these, 22 articles were excluded based on the full‐text screening and data extraction (see Figure 1 for reasons), and 12 eligible studies^22^, ^23^, ^24^, ^25^, ^26^, ^27^, ^28^, ^29^, ^30^, ^31^, ^32^, ^33^ involving 2439 cancer patients were included in the final analysis.

### 3.1 The overall effect

The overall effect on survival favored treatment groups, with an HR of 0.71 (95% Cl 0.58‐0.88; *P* = 0.001) and an NNT of 5.9, which suggests a small to moderate effect (Figure 2). The funnel plot was symmetric, although one study was stood out from the others. A sensitivity analysis was performed and exclusion of any single study did not change the overall results, which ranged from 0.68 (95% Cl 0.56‐0.82; *P* = 0.001) to 0.73 (95% Cl 0.59‐0.91; *P* = 0.003).

A moderate level of heterogeneity was detected among these studies according to Cochrane's Q (*χ* ^2^ = 29.3, *df* = 11) and *I* ^2 ^index (*P* = 0.002, *I* ^2^ = 62.6%). These results motivate the exploration of various subgroupings suggested by factors that might be important in affecting treatment effect on survival outcome.

### 3.2 Sources of heterogeneity

#### 3.2.1 Marital status

The median percentage of married patients over all studies was 66% and we used this as a cut‐off for categorization of studies. We categorized studies as having above median (*M* > 66%) or below/equivalent median (*M* ≤ 66%) percentage marital status. Seven studies were included in the category of above median marital status,^22^, ^25^, ^28^, ^30^, ^33^ and five studies were included in the category of below median marital status.^23^, ^24^, ^26^, ^27^, ^29^ Studies with below/equivalent median marital status (*M* ≤ 66%) showed a significant intervention effect on survival with an HR of 0.63 (95% 0.54‐0.74; *P *< 0.001) and an NNT of 4.5 (a moderate effect size by Cohen's Standards). On the other hand, studies above median (*M* > 66%) did not show a significant effect on survival with a HR of 0.89 (95% 0.74‐1.06; *P* = 0.19) and an NN of 28.6. Finally, comparison between studies reporting low vs high percentage married showed a statistically significant difference in effect (*z* = 2.75, *P* = 0.005) (Figure 3A).

#### 3.2.2 Intervention characteristics

Four studies used CBT as the intervention type, of which two reported its use in earlier cancer stages (Table 1)^29^, ^33^ and two of them in later stage.^23^, ^24^ Three studies used SEGT, three just supportive interventions, and two psychoeducational interventions.

**Table 1:** Descriptive summary of selected studies (1980‐2018)

Author	Sample				Intervention			Duration		Outcome
	No. of patients in I and C	Type and stage of cancer	Age, y (*M* ± SD or %)	Married (%)	Type	Format	Dose (h)	Intervention	Follow‐up (*M*)	Survival = HR (95% Cl)
Spiegel (1989)	I = 50 C = 36	Breast, metastatic	54.6 ± 10.2	62.5	SEGT	Group	78	1 y	10 y	0.51 (0.31, 0.83)
Cunningham (1998)	I = 30 C = 36	Breast, metastatic	49.5 ± 13	74	CBT	Group	84	35 wk	5 y	0.76 (0.43, 1.35)
Edelman (1999)	I = 43 C = 49	Breast, metastatic	50 ± 8.5	70.5	CBT	Group	50	6 mo	5 y	1.32 (0.84, 2.08)
McCorkle (2000)	I = 190 C = 185	Various types in various stages	≥65 = 66%	65.1	Supportive	Individual	12	4 wk	4 y	0.49 (0.32, 0.74)
Goodwin (2001)	I = 158 C = 77	Breast, metastatic	49.5 ± 10.3	71.6	SEGT	Group	78	1 y	7 y	1.23 (0.88, 1.72)
Kuchler (2007)	I = 136 C = 135	Gastrointestinal in various stages	56.8 ± 10.6	71.2	Psychoeducational	Individual	≥ every second day	During hospital stay	10 y	0.69 (0.52, 0.92)
Spiegel (2007)	I = 64 C = 61	Breast, metastatic	53.1 ± 10.6	57	SEGT	Group	78	1 y	14 y	0.93 (0.62, 1.40)
Andersen (2008)	I = 114 C = 113	Breast, stage II and III	≥ 50 = 51%	73.5	CBT	Group	39	1 y	11 y	0.44 (0.22, 0.86)
Bakitas (2009)	I = 161 C = 161	Various types with approximately 1 y	64.7 ± 10.8	65.2	Psychoeducational	Group	≥43	36 mo	15 mo	0.67 (0.50, 0.91)
Ross (2009)	I = 125 C = 124	Colorectal in different stages	68.8	60.5	Supportive	Individual	15	10 visits	8 y	0.68 (0.42, 1.09)
Temel (2010)	I = 77 C = 74	Non‐small‐cell lung, metastatic	64.9 ± 9.7	61.5	Supportive	Individual	Till the end	12 wk	6 mo	0.58 (0.40, 0.87)
Stagl (2015)	I = 120 C = 120	Breast, 0‐IIIB	49.7 ± 9.0	62.5	CBT	Group	15	10 wk	11 y	0.21 (0.05, 0.93)

C, control group; CBT, cognitive‐behavioral therapy; I, intervention group; SEGT, supportive‐expressive group therapy.

Studies with CBT that sampled patients with early‐stage cancer showed a significant effect on survival with an HR of 0.39 (95% 0.21‐0.72; *P *< 0.001) and an NNT of 2.6 (a large effect size) (Figure 3B). By contrast, in studies using CBT among patients with late‐stage cancer, the analysis showed no statistically significant effect on survival (HR = 1.1, 95% 0.75‐1.53; *P* = 0.7) and an NNT of −28.6. When comparing early vs late in CBT, a *z*‐test showed a significant survival effect favoring studies with a CBT that sampled patients with early stage (*z* = 2.46, *P* = 0.01).

#### 3.2.3 Age

We categorized studies into three groups: younger (mean age *M* < 50 years),^23^, ^24^, ^26^, ^33^ middle‐aged (50 years < *M* < 60 years),^22^, ^27^, ^28^, ^29^ and older (*M* > 60 years).^25^, ^30^, ^31^, ^32^ Among the studies that involved younger patients, intervention did not significantly affect survival (HR = 1.1, 95% 0.87‐1.40; *P* = 0.43), with an NNT = −20.1 (Figure 3C). However, in studies sampling both those aged between 50‐60 and above 60 years, analysis showed a significant survival result favoring intervention (HR = 0.68, 95% 0.55‐0.83; *P *< 0.001 and HR = 0.61, 95% 0.50‐0.74; *P *< 0.001, respectively), with an NNT of 5.3 (small to moderate effect) for studies sampling 50‐60‐year‐old patients and an NNT of 4.2 (medium effect) for studies sampling patients older than 60 years. A statistical comparison was made between studies sampling younger (>50 years) and older subjects (aged above 60 years) and comparison confirmed that intervention for studies sampling older cancer patients seemed more beneficial (*z* = 2.5, *P* = 0.01). Similar results can be observed when comparing younger vs middle‐aged (50‐60 years old).

#### 3.2.4 Clinical characteristics

Most of the studies sampled breast cancer patients (58%), and of those, the majority was comprised of MBC (71%). One study included metastatic lung cancer patients, one colorectal, one gastrointestinal, and one included various cancer types. There were not enough studies on any one cancer type to assess this as a source of heterogeneity.

In summary, major sources of heterogeneity among studies appear to be (a) marital status (studies with below/equivalent median percentage of married participant respond better), (b) treatment interaction with cancer stage (within the CBT group, those with early‐stage cancer respond better), and (c) the age of the patients (older patients respond better).

## 4 DISCUSSION

Although the focus of this paper is to seek and report clues that emerged from sources of heterogeneity, we note that in the 12 studies that we believe are methodologically strong enough to be included, psychosocial intervention is shown to have overall a small to moderate effect on survival. This finding is in accordance with a meta‐analysis published by three studies,^4^, ^8^, ^10^ but not with five others.^3^, ^5^, ^6^, ^7^, ^9^

Inconclusive results in those five studies are in part the result of inappropriate interpretation of “statistical significance” or “*P*‐values”. A statistically significant result (generally *P *< 0.05) means that the design was good enough, the sample size large enough, outcome measure reliably enough measured, in short, the power was adequate to detect some deviation from the null hypothesis, here of treatment vs control equivalence. It does not necessarily mean that the treatment is meaningfully better than the control condition. If a result is not statistically significant, the confidence interval for the effect size will include zero, and the results are inconclusive. It certainly does not tell us that the treatment is not effective. How much of the effect there is, how clinically important the results are, is conveyed with an effect size, a population parameter that can be interpreted in terms of clinical significance.

Another reason for previous inconclusive results might be the effect size used. Most of the previous meta‐analyses that did not find an effect on survival used OR or RR as an effect size. Both focus on only one fixed follow‐up time (eg, 1 or 2 or 5 years), thus drawing inferences from that one point about the entire curve. Knowing the RR or OR at one point of time does not unequivocally support or refute the overall superiority of either treatment, much less the clinical significance of any treatment effect. Single time point effect sizes such as RR and OR should not be used as the effect sizes in meta‐analysis comparing survival.

The hazard ratio as an effect size was used by two studies and both of those studies reported no overall effect.^3^, ^9^ However, one of those two meta‐analyses^3^ involved only two studies that exhibited substantial heterogeneity. The other meta‐analysis^9^ included a much larger number of RCTs (15 studies), but some of those included studies used different types of control condition, for example relaxation classes, home study cognitive‐behavioral package. This complicates comparability across studies. Our hope was that, by removing the “garbage in, garbage out” problems in Step 1, and by reducing the “apples and oranges” problems in Steps 2 and 3, the avoidable inconsistencies seen in previous meta‐analysis will be reduced, and the remaining will suggest true sources of heterogeneity of effect sizes.

In meta‐analysis, the most important conclusion is the quantitative summary of the results. However, when meta‐analysis includes studies that cover populations that differ from one another in demographic and clinical characteristics, along with variation in the nature of the interventions, the goal should be to evaluate those differences.^34^ Here we explored several potentials sources and found three: marital status, cancer stage of those undergoing CBT intervention, and age. This suggests strongly that in any future RCTs, these three factors might be considered as possible moderators of treatment outcome, and thus as indications to patients and clinicians regarding who is most likely to benefit from psychosocial interventions.

Psychosocial intervention appears to be more effective in studies with a lower percentage married (≤66%) (see Figure 3A). This finding makes sense in that psychotherapy provides social and emotional contact and support that is more likely to be a problem among unmarried individuals. Indeed, there is strong evidence that marital status is an independent predictor of cancer survival. In a large five‐year study using the SEER registry involving 734 889 patients, married cancer patients lived an average of four months longer than unmarried cancer patients with all ten cancer types studied. The authors noted that this was comparable to the overall effects of chemotherapy on survival.^35^ So the married subgroup may both have less need for emotional support and have already benefitted from any psychosocial effect on survival.

A second identified moderator was found in studies employing CBT treatment and applies to the cancer stage. Those studies sampling cancer patients in the early stage show greater effect sizes from CBT intervention (See Figure 3B). During the cancer trajectory, patients go through various phases. In the early stage, patients may experience acute anxiety, grief, and anger, while in the later phases patients need to be involved in the process of dying and working through what it is like to be more imminently facing death (ie, “detoxifying” death),^36^, ^37^ not merely on reducing symptoms. The practical, symptom‐management approach of CBT may well have more profound effects among more recently diagnosed patients, while the emotionally expressive existential focus of SEGT may be more helpful in the later stages of progressive disease. Thus, the phase of the disease may be a critical factor in determining which psychosocial intervention should be recommended.

Third, our analysis suggested that studies that sampled patients that were on average younger than 50 years, showed smaller effect sizes (See Figure 3C). Younger patients are often considered to have a more aggressive cancer type and are often treated differently, with more radical surgeries, as well as more adjuvant treatment such as radiation and chemotherapy.^38^ Treatment with chemotherapy often leads to menopausal symptoms, weight gain, hair loss, and other treatment‐related problems that can have a profound negative effect on the quality of life.^39^ Additional stress may be caused by their restricted activities and higher expectations about functional status and less experience with illness and disability.^40^ Younger patients have reported feeling isolated from other older cancer patients in support groups.^41^ If during an intervention their unique needs aren't properly addressed, younger cancer patients might be less motivated to actively participate in psychosocial interventions and thus, benefit less. An age‐appropriate intervention to better address age‐specific needs might be recommended.

Although this paper has focused on the clinical sources of heterogeneity, it is important to mention that statistical heterogeneity can also be caused by poor methodological quality or publication bias.^34^ Some of the included studies, while valid, suffer from the lack of power (as seen with very wide confidence intervals). For example, Stagl et al^33^ reported one of the most positive results (HR = 0.31) with one of the largest sample size (120, 120), but with an extremely wide confidence interval (see Figure 3) (indicating limited power), probably the result of having only 14% mortality over 11 years. Power in survival comparisons depends not only on the total sample size but on the duration of follow‐up and on the number of deaths observed in the two groups.

There are some strengths and limitations here to be noted when interpreting the results. An important strength is a search strategy that was based on four databases and included a backwards organic search of the previous meta‐analyses. Moreover, the inclusion/exclusion criteria were based on reviewing those past meta‐analyses and discussing limitations that may cause confusion in their overall conclusions. Additionally, we have reported Number Needed to Treat, as NNT is easier to understand for clinicians and patients and it reflects clinical significance more clearly than does HR A major strength was a careful investigation of potential sources of heterogeneity. However, most of our analyses were based on a small number of trials precluding drawing definitive conclusions. For this reason and its exploratory nature, the results of this paper are considered only as a rationale and justification for hypotheses to be considered in future research.

From a research point of view, we urge that future meta‐analyses take heterogeneity more seriously, not just observing its presence. Identifying factors that contribute to heterogeneity will allow future investigators to design studies with populations of patients more likely to respond, thereby increasing the effect size and the precision of the effect size estimation, reducing the required sample size necessary for adequate power to test the hypothesis. From a clinical point of view, perhaps one of the most important findings that emerged here is increased awareness of the importance of considering demographic and clinical characteristics of the patients when recommending an individual patient to a specific psychosocial intervention. Patients should be treated according to their needs, and their ability to respond to interventions of specific types. If so, psychosocial intervention may be found to influence not merely the quality of life, but also the quantity of life. For some people with cancer, living better can also mean living longer.

## CONFLICT OF INTEREST

None declared.
