# Supplementary material for: “Not just another meta‐analysis”: Sources of heterogeneity in psychosocial treatment effect on cancer survival
Source: Cancer Med. 2019 Jan 1;8(1):363–73. doi: 10.1002/cam4.1895 (PMC6346264; doi:10.1002/cam4.1895)
Supplement: Supplementary file 1 [file CAM4-8-363-s001.doc]

| **Steps** | **Ovid MEDLINE^1^** | **PubMed^2^** | **PsycINFO^3^** | **Cochrane Central Register of Controlled Trials^4^** |
| --- | --- | --- | --- | --- |
| Step 1: Find population: cancer patients |  |  |  |  |
| Search strategy | cancer.tw. OR neoplasm.tw. OR oncology.tw. OR carcinoma.tw. OR lymphoma.tw. OR melanoma.tw. OR leukemia.tw. OR sarcoma.tw. OR cancer.mp. OR neoplasm.mp. OR oncology.mp. OR carcinoma.mp. OR lymphoma.mp. OR melanoma.mp. OR leukemia.mp. OR sarcoma.mp. | cancer[Title/Abstract] OR neoplasm[Title/Abstract] OR oncology[Title/Abstract] OR carcinoma [Title/Abstract] OR lymphoma [Title/Abstract] OR melanoma [Title/Abstract] OR leukemia [Title/Abstract] OR sarcoma [Title/Abstract] | cancer* OR neoplasm* OR oncology OR carcinoma OR lymphoma OR melanoma OR leukemia OR sarcoma | (cancer):ti,ab,kw OR (neoplasm):ti,ab,kw OR (oncology):ti,ab,kw OR (carcinoma):ti,ab,kw OR (lymphoma):ti,ab,kw OR (melanoma):ti,ab,kw OR (leukemia):ti,ab,kw OR (sarcoma):ti,ab,kw |
| Hits | 2,565,519 | 2,124,086 | 2,461 | 120,207 |
| Step 2: Find treatment: psychosocial interventions |  |  |  |  |
| Search strategy | behavior therapy.tw. OR behavior therapy.tw. OR cognitive therapy.tw. OR counselling.tw OR supportive-expressive therapy.tw. OR group psychotherapy.tw. OR group support.tw. OR group therapy.tw. OR meditation.tw. OR mindfulness-based.tw. OR nursing intervention.tw. OR nursing support.tw. OR patient education.tw. OR psychoanalytic therapy.tw. OR psychoeducational therapy.tw. OR supportive therapy.tw. OR psychological intervention.tw. OR psychological treatment.tw. OR psychosocial intervention.tw. OR psychosocial support.tw. OR psychosocial treatment.tw. OR psychotherapy.tw. OR support group.tw. OR support groups.tw. | behavior therapy[Title/Abstract] OR behavior therapy [Title/Abstract] OR cognitive therapy [Title/Abstract] OR counselling [Title/Abstract] OR supportive-expressive therapy [Title/Abstract] OR group psychotherapy [Title/Abstract] OR group support [Title/Abstract] OR group therapy [Title/Abstract] OR meditation [Title/Abstract] OR mindfulness-based [Title/Abstract] OR nursing intervention [Title/Abstract] OR nursing support [Title/Abstract] OR patient education [Title/Abstract] OR psychoanalytic therapy [Title/Abstract] OR psychoeducational therapy [Title/Abstract] OR supportive therapy [Title/Abstract] OR psychological intervention [Title/Abstract] OR psychological treatment [Title/Abstract] OR psychosocial intervention [Title/Abstract] OR psychosocial support [Title/Abstract] OR psychosocial treatment [Title/Abstract] OR psychotherapy [Title/Abstract] OR support group [Title/Abstract] OR support groups [Title/Abstract] | behavior therapy OR behavior therapy OR cognitive therapy OR counselling OR supportive-expressive therapy OR group psychotherapy OR group support OR group therapy OR meditation* OR mindfulness-based OR nursing intervention* OR nursing support OR patient education* OR psychoanalytic therapy OR psychoeducational therapy OR supportive therapy OR psychological intervention* OR psychological treatment* OR psychosocial intervention* OR psychosocial support OR psychosocial treatment* OR psychotherapy OR support group* | (behavior therapy):ti,ab,kw OR (behaviour therapy):ti,ab,kw OR (supportive-expressive therapy):ti,ab,kw OR (coping skills training):ti,ab,kw OR (group psychotherapy):ti,ab,kw OR (counselling therapy):ti,ab,kw OR (counseling therapy):ti,ab,kw OR (psychoanalytic therapy):ti,ab,kw OR (meditation):ti,ab,kw OR (support group):ti,ab,kw OR (psychoeducational therapy):ti,ab,kw OR (patient education):ti,ab,kw OR (client education):ti,ab,kw OR (nursing intervention):ti,ab,kw OR (nursing support):ti,ab,kw OR (psychotherapy):ti,ab,kw OR (psychosocial treatment):ti,ab,kw OR (psychological treatment):ti,ab,kw OR (psychosocial intervention):ti,ab,kw OR (psychological intervention):ti,ab,kw OR (psychosocial support):ti,ab,kw OR (cognitive therapy):ti,ab,kw |
| Hits | 990,050 | 102,024 | 25,514 | 89,882 |
| Step 3: Find outcome: survival |  |  |  |  |
| Search strategy | survival OR mortality OR survival analysis OR survival rate OR Kaplan-Meier Estimate OR disease-free survival | survival OR mortality[Title/Abstract] OR survival analysis[Title/Abstract] OR survival rate[Title/Abstract] OR Kaplan-Meier Estimate OR disease-free survival | survival OR mortality OR survival analysis OR survival rate* OR disease-free survival | survival OR mortality OR survival analysis OR survival rate OR Kaplan-Meier Estimate OR disease-free survival |
| Hits | 1,299,530 | 1,043,290 | 9,566 | 110,316 |
| Step 4: Find design: randomized-controlled trials |  |  |  |  |
| Search strategy | clinical trial OR randomized OR randomly OR trial OR groups | (randomized controlled trial OR controlled clinical trial OR randomized[tiab] OR clinical trials as topic[mesh:noexp] OR randomly[tiab] OR trial[ti] NOT (animals[mh] NOT humans [mh])) | random* OR control* OR SU.EXACT.EXPLODE (treatment) | clinical trial* OR randomized OR trial* OR randomized clinical OR controlled clinical trial* OR randomized (in title, abstract, keywords in trials) |
| Hits | 4,203,280 | 978,593 | 128,489 | 48,515 |
| Step 5: Combined search (1-4) |  |  |  |  |
| Search strategy | #1 AND #2 AND #3 AND #4 | #1 AND #2 AND #3 AND #4 | #1 AND #2 AND #3 AND #4 | #1 AND #2 AND #3 AND #4 |
| Hits | 1667 | 207 | 2 | 1770 (only trials) |

^1^ <http://cmk-proxy.mf.uni-lj.si:2062/sp-3.25.0a/ovidweb.cgi>

^2^ <https://www.ncbi.nlm.nih.gov/pubmed/>

^3^ [http://cmk-proxy.mf.uni-lj.si:2994/ehost/search/basic?sid=9bf24f69-7f58-41f5-9e0a-18c603de8e75%40sessionmgr4007&vid=2&hid=4207](http://cmk-proxy.mf.uni-lj.si:2994/ehost/search/basic?sid=9bf24f69-7f58-41f5-9e0a-18c603de8e75@sessionmgr4007&vid=2&hid=4207)

^4^ <http://onlinelibrary.wiley.com/cochranelibrary/search>
